# Clinical outcomes among individuals with systemic inflammation following myocardial infarction: Insights from the veterans affairs healthcare system

**DOI:** 10.1016/j.ajpc.2026.101657

**Published:** 2026-04-30

**Authors:** Alexander T Sandhu, Adam Furst, Fatima Rodriguez, Neil Kalwani, David J. Maron, Shriram Nallamshetty, Natasha Din, Michael Fan, Kat Khachatourian, Jeffrey R. Skaar, Ivy Tonnu-Mihara

**Affiliations:** a Greater Los Angeles Veterans Affairs Healthcare System, Los Angeles, CA, USA; b University of California Los Angeles, Los Angeles, CA, USA; c Palo Alto Veterans Affairs Healthcare System, Palo Alto, CA, USA; d Division of Cardiology, Stanford University School of Medicine, Stanford, CA, USA; e Stanford Prevention Research Center, Stanford University School of Medicine, Stanford, CA, USA; f Medical Affairs, Novo Nordisk Inc., Plainsboro, NJ, USA; g Clinical Data Science and Evidence, Novo Nordisk Inc., Plainsboro, NJ, USA

**Keywords:** Inflammation, Coronary artery disease, Myocardial infarction, High-sensitivity C-reactive protein, Health resources

## 1 Background

Individuals with a prior myocardial infarction (MI) are at high risk of subsequent cardiovascular complications. A growing body of evidence highlights the role of systemic inflammation (SI) in this elevated risk [1,2]. MI events contribute to acute SI via increased inflammatory cytokines and activation of immune cells [1,2]. For many, this results in chronic SI that drives progressive atherosclerosis and plaque instability. In this analysis, we evaluated the association between SI and clinical and economic outcomes following an MI in a national cohort of Veterans between 2008–2022.

## 2 Methods

We used VHA electronic health record (EHR) data and administrative claims from Veterans’ purchased care and Medicare, including demographics, encounters, diagnoses, procedures, laboratory results, vital signs, and pharmacy records. Mortality data were based on the VHA Mortality Data Repository with underlying cause of death available through 2022.

We identified Veterans with an outpatient hsCRP measurement post-MI from 2008–2022. We identified MI hospitalizations based on ICD-9/ICD-10 principal diagnosis codes. The first outpatient hsCRP measurement within 60 to 730 days post-MI was set as the index date. We excluded hsCRP in the first 60 days post-MI to exclude a transient increase in SI.

To identify a cohort with active VHA care, we excluded Veterans without at least 1 diagnosis and encounter in the year before hsCRP measurement. Finally, we excluded individuals with comorbidities known to increase SI, adverse cardiovascular outcomes, and utilization: end-stage renal disease, severe hepatic disease, HIV, metastatic cancer or cancer receiving chemotherapy, and chronic osteomyelitis to provide a conservative estimate of the association between hsCRP and outcomes.

For the primary analysis, we defined SI as hsCRP levels ≥2 mg/L and ≤10 mg/L based on prior analyses [3]. We excluded hsCRP >10 mg/L because marked elevation of hsCRP may be related to acute infections or other acute systemic disorders with increased risk of adverse clinical outcomes [2]. We captured sociodemographics, vital signs, comorbidities, laboratory values, and medications, and defined baseline characteristics based on a 3-year lookback period.

Our primary clinical outcome was a 3-component major adverse cardiovascular event (MACE) composite: all-cause death, MI, or stroke. MI and stroke were identified based on the principal diagnosis. Secondary outcomes included a 5-component MACE: all-cause death, MI, stroke, coronary revascularization, and HF hospitalization; each individual component of the 5-point MACE; and cardiovascular death. Revascularization was identified based on procedural codes. Cardiovascular death ascertainment was based on underlying cause of death ICD-10 code for cardiovascular disease (I.00-I.99). We identified outcomes post-hsCRP measurement (the index date).

For acute care utilization, we captured the frequency of all-cause hospitalizations, cardiovascular hospitalizations, and HF hospitalizations. We identified cardiovascular hospitalizations based on the principal diagnosis. We calculated utilization as the average per patient-year of follow-up.

For costs, we analyzed the following cost categories: total, inpatient, outpatient, pharmacy, and cardiovascular-related. Cardiovascular-related costs were identified based on an associated ICD code consistent with cardiovascular disease. VHA costs were based on average cost methods in which services are weighted by Medicare relative value weights and adjusted to the total VHA cost of care. Medicare and community care costs used the total amount paid. All costs were inflated to 2023 US dollars.

For clinical outcomes, we evaluated the adjusted association between SI and outcomes using Cox survival models. We censored events on the date of last known encounter. We assumed the hazard ratios were the average of time-varying hazard ratios. For non-fatal events, we censored outcomes on the date of death. We modeled the association between SI and healthcare resource utilization using negative binomial regression. We modeled the association between SI and costs via gamma regression models, with the results displayed as the average marginal cost associated with SI while excluding censored individuals. Given inpatient and cardiovascular costs are often zero, we modeled these categories via two-stage models. We adjusted for age, sex, year of hsCRP measurement, the Charlson comorbidity index, prior MI, prior coronary revascularization, smoking status, body mass index, eGFR, and secondary prevention medications known to improve outcomes among patients with ASCVD (i.e.*,* lipid-lowering therapies, antiplatelets, SGLT2 inhibitors, and GLP-1 receptor agonists). Continuous variables were modeled as a restricted cubic spline with four knots. Given low missingness (<3%), we used mean imputation with missing indicators to account for non-random missingness.

## 3 Results

Between 2008–2022, we identified 13,090 Veterans with hsCRP measured 60–730 days post-MI. After applying exclusion criteria, there were 11,230 Veterans (85.8%), including 7956 Veterans with hsCRP ≤10 mg/L; 4594 Veterans (57.7%) with SI (hsCRP ≥2 mg/L); and 3362 Veterans (42.3%) without SI (hsCRP <2 mg/L). The median year of hsCRP measurement was 2015 (IQR: 2012–2018).

Veterans with SI were slightly younger than those without SI (67.4 years [SD 9.6] vs. 67.9 years [SD 9.9], p = 0.04). A similar proportion were women in both cohorts (3.4% vs. 2.9%, p = 0.24). Veterans with SI were less likely to have prior STEMI (25.2% vs. 28.9%, p < 0.01) or have undergone coronary revascularization (54.7% vs. 56.9%, p = 0.035). The median time between MI and hsCRP was similar (302 days [IQR: 154–495] vs. 309 days [IQR: 162–507], p = 0.14). Veterans with SI were more likely to have chronic kidney disease (14.0% vs. 10.6%, p < 0.001), diabetes mellitus (41.1% vs. 33.0%, p < 0.001), heart failure (35.3% vs. 27.0%, p < 0.001), and systemic autoimmune disease (14.5% vs. 12.6%, p = 0.02), while the prevalence of liver disease (6.6% vs. 6.9%, p = 0.59) was similar.

Veterans with SI had higher body mass index (30.8 [SD: 5.9] vs. 29.2 [SD: 4.9], p < 0.001) but similar systolic blood pressure (136.5 mmHg [SD: 13.5] vs. 136.1 [SD: 13.1], p = 0.12). Veterans with SI had slightly higher hemoglobin A1c (6.8 [SD: 1.5] vs. 6.5 [SD: 1.5]), p < 0.001). Both groups had similar use of lipid-lowering therapy (73.7% vs. 72.8%, p = 0.38) and LDL-cholesterol (104mg/dL [SD: 37] vs. 103mg/dL [SD: 35], p = 0.21).

The 3-point MACE outcome rate was 10.3 (95% CI: 9.9–10.7) per 100 patient-years (100PY) among those with SI compared with 7.6 (95% CI: 7.2–8.0) among those without SI (p < 0.001) over a median of 4.4 years (Table 1). After adjustment, the HR of SI was 1.28 (95% CI: 1.19–1.37). For 5-point MACE, SI was associated with a 21% increased hazard (HR: 1.21; 95% CI: 1.14–1.29). SI was also significantly associated with all-cause death, MI, and peripheral arterial revascularization (Table 1). However, no association was observed between SI and stroke or coronary revascularization risk. After adjustment, there was a 26% greater risk of cardiovascular death among those with SI (HR: 1.26; 95% CI: 1.12–1.41).

**Table 1:** Association between hsCRP level (stratified at >2mg/L and <2mg/L) and clinical outcomes, healthcare utilization, and cost of care.

Clinical Outcomes	Event Rates (Per 100 PY [95% CI])		P-value^†^	Adjusted HR
	hsCRP≥2 and ≤10 (n = 4594)	hsCRP<2 (n = 3362)		
3-point MACE	10.3	7.6	<0.001	1.28*
	(9.9 – 10.7)	(7.2 – 8.0)		(1.19 - 1.37)
5-point MACE	14.4	10.9	<0.001	1.21*
	(13.8 – 14.9)	(10.4 – 11.5)		(1.14 - 1.29)
All-cause death	8.4	6.2	<0.001	1.30*
	(8.1 – 8.8)	(5.8 – 6.5)		(1.21 - 1.40)
Cardiovascular death	3.1	2.3	<0.001	1.26*
	(2.9 - 3.4)	(2.1–2.6)		(1.12 - 1.41)
Stroke (ischemic stroke or TIA)	0.6	0.5	0.391	0.99
	(0.5 - 0.7)	(0.4 - 0.6)		(0.76–1.30)
Acute myocardial infarction	1.8	1.3	<0.001	1.27*
	(1.7 – 2.0)	(1.2 – 1.5)		(1.08 - 1.49)
HF hospitalization	1.9	1.1	<0.001	1.42*
	(1.7 - 2.1)	(0.9 - 1.2)		(1.20 - 1.69)
Coronary revascularization	5.4	4.5	<0.001	1.09
	(5.1 – 5.7)	(4.2 – 4.9)		(0.99 – 1.20)
Peripheral revascularization	2.7	2.0	<0.001	1.22*
	(2.5 – 3.0)	(1.8 – 2.2)		(1.06 – 1.39)
Healthcare Utilization	Event Rates (Per 100PY [95% CI])		P-value^†^	Adjusted IRR
	hsCRP≥2 and ≤10 (n = 4594)	hsCRP<2 (n = 3362)		
All Cause Hospitalization	80.9	67.4	<0.001	1.23*
	(79.9–81.9)	(66.4–68.5)		(1.17 - 1.30)
Cardiovascular Hospitalization	27.3	21.6	<0.001	1.15*
	(26.8–28.0)	(21.0–22.2)		(1.07- 1.23)
MI Hospitalization	2.6	2.0	0.0234	1.19*
	(2.4–2.8)	(1.9–2.2)		(1.01 - 1.39)
HF Hospitalization	3.9	2.3	<0.001	1.44*
	(3.7–4.1)	(2.1–2.5)		(1.17- 1.76)
Healthcare Costs	Costs Per PY of Follow-up (Median [IQR])^⁎⁎^		P-value^†^	Marginal Cost of SI Over 1 Year**
	hsCRP≥2 and ≤10 (n = 4594)	hsCRP<2 (n = 3362)		
Total	33,520	26,402	<0.001	7470^†^
	(16,962 - 66,311)	(13,263 - 51,022)		(4887 - 10,052)
Inpatient	10,203	6459	<0.001	5351^†^
	(1531 - 31,586)	(238 - 20,641)		(3269 - 7433)
Cardiovascular	7112	4885	<0.001	3182^†^
	(2142 - 20,060)	(1591 - 14,181)		(1476 - 4888)
Outpatient	16,406	14,047	<0.001	2323^†^
	(9337 - 28,080)	(8171 - 24,210)		(1292 - 3354)
Pharmacy	1804	1401	<0.001	87
	(812 - 3673)	(579 - 3159)		(−210 - 383)

*Abbreviations: HR: hazard ratio; IRR: incidence rate ratio; hsCRP: high-sensitivity C-reactive protein; MACE: major adverse cardiovascular outcomes; SI: systemic inflammation; TIA: transient ischemic attack.

^†^The p-value for the difference in unadjusted rates or costs between Veterans with SI and without SI.

^§^Adjusted for age, sex, year of hsCRP measurement, Charlson comorbidity index, prior MI, prior coronary revascularization, smoking status, body mass index, GFR, lipid-lowering therapies, antiplatelets, SGLT2i, and GLP-1 RA.

^‖^3-point MACE includes all-cause death, non-fatal MI, and non-fatal stroke.

^#^5-point MACE includes all-cause death, non-fatal MI, non-fatal stroke, coronary revascularization, and HF hospitalization.

**Costs in 2023 US dollars.

We found higher acute care utilization among Veterans with SI (Table 1). After adjustment, SI was associated with a 23% increase in the rate of all-cause hospitalizations (IRR: 1.23; 95% CI: 1.17–1.30) and a 15% increase in cardiovascular hospitalizations (IRR: 1.15; 95% CI: 1.07–1.23).

We found higher healthcare costs among Veterans with SI. Over 1 year of follow-up, SI was associated with $7470 (95% CI: $4887-$10,052) in additional total costs and $5351 (95% CI: $3269-$7433) in inpatient costs after adjustment. For cardiovascular-related costs, SI was associated with $3182 (95% CI: $1476-$4888) in additional costs. Costs were also significantly greater for pharmacy and outpatient costs (Table 1).

## 4 Discussion

In a national cohort of Veterans with recent MI, SI was associated with increased risk of recurrent major cardiovascular events and increased healthcare utilization and costs. Among Veterans with prior MI, 70% had evidence of SI. Veterans with and without SI had relatively similar age, risk burden, and treatment with lipid-lowering therapies. However, those with SI had substantially higher risk of adverse outcomes. Our results extend prior findings from randomized trials to a real-world cohort.

These results add to a growing literature suggesting the value of hsCRP to stratify the risk of those with known ASCVD. The 2024 ESC Guidelines for Chronic Coronary Syndrome recommend hsCRP testing to improve risk stratification [4]. Furthermore, the SMART2 secondary prevention risk score includes hsCRP levels in risk prediction [4]. A 2025 ACC Scientific Statement also recommends universal screening for hsCRP among primary and secondary ASCVD prevention patients [2]. Evaluation for chronic SI can improve risk stratification post-MI to guide shared decision-making regarding cardiovascular prevention. While prior trials of anti-inflammatory therapies for preventing recurrent ASCVD events have had mixed results, ongoing trials are investigating novel anti-inflammatory therapies.

There are multiple limitations to this study. First, there may be unmeasured variables that confound the association between hsCRP levels and outcomes. This is relevant given Veterans with SI had higher rates of select comorbidities that may contribute to outcome differences. Second, the decision to measure hsCRP may reflect additional clinical concern that may increase cohort risk, although a prior analysis found similar characteristics among patients that underwent hsCRP testing [5]. Third, the use of non-adjudicated outcomes may introduce event misclassification. Fourth, our historical cohort has low rates of GLP1-RA and SGLT2i treatment. These therapies have indirect effects on inflammation and may modify inflammatory risk following MI. Finally, our cohort is predominantly male with non-Hispanic ethnicity, which may limit the generalizability of the results.

Our analysis demonstrates the increased risk of adverse cardiovascular events and costs among Veterans with prior MI and SI. These findings suggest the importance of recognizing SI as a marker of increased risk among patients with prior MI.

## Funding

This work was funded by 10.13039/501100004191Novo Nordisk. This material is the result of work supported with resources and the use of facilities at the VA Palo Alto Healthcare System, Palo Alto, CA and the Greater Los Angeles VA Healthcare System, Los Angeles, CA. Support for the Department of Veterans Affairs (VA) and Centers for Medicare and Medicaid Services data was provided by the VA and the VA Information Resource Center. The funders contributed to the design of the study, interpretation of the data, and review of the manuscript. The funders had no role in the collection, management, analysis, or the decision to submit the manuscript for publication.

## CRediT authorship contribution statement

**Alexander T Sandhu:** Writing – original draft, Supervision, Methodology, Investigation, Formal analysis, Data curation, Conceptualization. **Adam Furst:** Writing – review & editing, Methodology, Investigation, Formal analysis. **Fatima Rodriguez:** Writing – review & editing, Investigation. **Neil Kalwani:** Writing – review & editing, Investigation. **David J. Maron:** Writing – review & editing, Investigation. **Shriram Nallamshetty:** Writing – review & editing, Investigation. **Natasha Din:** Writing – review & editing, Investigation. **Michael Fan:** Writing – review & editing, Investigation, Conceptualization. **Kat Khachatourian:** Writing – review & editing, Methodology, Investigation, Conceptualization. **Jeffrey R. Skaar:** Writing – review & editing, Methodology, Investigation, Conceptualization. **Ivy Tonnu-Mihara:** Writing – review & editing, Supervision, Investigation, Conceptualization.

## Declaration of competing interest

The authors declare the following financial interests/personal relationships which may be considered as potential competing interests:

Alexander Sandhu reports financial support was provided by Novo Nordisk Inc. Alexander Sandhu reports a relationship with 10.13039/100002429Amgen that includes: funding grants. Alexander Sandhu reports a relationship with Astra Zeneca that includes: funding grants. Alexander Sandhu reports a relationship with Bayer AG that includes: funding grants. Alexander Sandhu reports a relationship with 10.13039/100008272Novartis Pharmaceuticals Corporation that includes: funding grants. Alexander Sandhu reports a relationship with Reprieve Cardiovascular that includes: consulting or advisory. Alexander Sandhu reports a relationship with Holosis that includes: consulting or advisory. Alexander Sandhu reports a relationship with Cleerly that includes: consulting or advisory. Fatima Rodriguez reports a relationship with Carta Healthcare Inc that includes: equity or stocks. Fatima Rodriguez reports a relationship with HealthPals that includes: consulting or advisory and equity or stocks. Fatima Rodriguez reports a relationship with Novartis Pharmaceuticals Corporation that includes: consulting or advisory. Fatima Rodriguez reports a relationship with Novo Nordisk Inc that includes: consulting or advisory. Fatima Rodriguez reports a relationship with Esperion Therapeutics Inc that includes: consulting or advisory. Fatima Rodriguez reports a relationship with Movano Health that includes: consulting or advisory. Fatima Rodriguez reports a relationship with Kentho Health that includes: consulting or advisory. Fatima Rodriguez reports a relationship with Inclusive Health that includes: consulting or advisory. Fatima Rodriguez reports a relationship with Edwards Lifesciences Corporation that includes: consulting or advisory. Fatima Rodriguez reports a relationship with Cleerly Inc that includes: consulting or advisory. Fatima Rodriguez reports a relationship with Arrowhead Pharmaceuticals Inc that includes: consulting or advisory. Fatima Rodriguez reports a relationship with iRhythm Technologies Inc that includes: consulting or advisory. Fatima Rodriguez reports a relationship with HeartFlow Inc that includes: consulting or advisory. David Maron reports a relationship with Ablative Solutions Inc that includes: equity or stocks. David Maron reports a relationship with Preemptive AI that includes: equity or stocks. David Maron reports a relationship with JVMP Labs that includes: equity or stocks. David Maron reports a relationship with Cleerly Inc that includes: funding grants. David Maron reports a relationship with Omada Health that includes: funding grants. David Maron reports a relationship with HeartFlow Inc that includes: consulting or advisory. David Maron reports a relationship with Inno Med that includes: consulting or advisory. David Maron reports a relationship with Johnson and Johnson that includes: consulting or advisory. David Maron reports a relationship with Regeneron Pharmaceuticals Inc that includes: consulting or advisory. David Maron reports a relationship with SCILEX Pharmaceuticals that includes: consulting or advisory. David Maron reports a relationship with New Amsterdam School that includes: consulting or advisory. David Maron reports a relationship with Bayer AG that includes: consulting or advisory. Michael Fan reports a relationship with Novo Nordisk Inc that includes: employment. Kat Khachatourian reports a relationship with Novo Nordisk Inc that includes: employment and equity or stocks. Jeffrey R. Skaar reports a relationship with Novo Nordisk Inc that includes: employment and equity or stocks. Jeffrey R. Skaar reports a relationship with Trinity Life Science that includes: equity or stocks. Ivy Tonnu-Mihara reports a relationship with Novo Nordisk Inc that includes: employment. If there are other authors, they declare that they have no known competing financial interests or personal relationships that could have appeared to influence the work reported in this paper.
